# Road Dust, PM_10_ Exposure and Respiratory Health in Visby: An Updated Analysis of Mitigation Efforts

**DOI:** 10.3390/toxics14060497

**Published:** 2026-06-07

**Authors:** Henrik Olstrup, Bertil Forsberg, Andreas Tornevi

**Affiliations:** Department of Epidemiology and Global Health, Umeå University, Försörjningsvägen 7D, 907 37 Umeå, Sweden; bertil.forsberg@umu.se (B.F.); andreas.tornevi@umu.se (A.T.)

**Keywords:** air pollution, PM_10_, road dust, respiratory health, respiratory visits, short-term effects, limestone, crushed granite

## Abstract

**Background:** High concentrations of coarse particulate matter PM_10_ from road dust are a major air quality concern in Visby, Sweden. To mitigate these levels, local authorities replaced soft limestone with crushed granite as an anti-slip material starting in the winter of 2023/2024. This is a follow-up study evaluating the impact of this intervention on PM_10_ concentrations and the associated short-term respiratory health effects. **Methods:** Daily counts of healthcare visits for respiratory diseases (ICD-10: J00–J99) and daily mean PM_10_ concentrations were analyzed using a quasi-Poisson regression model. This study compared the limestone period (2015–2019) with the granite period (2023–2025), stratified by season (winter/spring and summer/autumn) and age group (children 0–17 years and adults >17 years). **Results:** The transition to crushed granite reduced peak PM_10_ concentrations during the spring. For adults, the relative risks for respiratory visits during winter/spring decreased during the granite period when compared to the limestone period (Wald *p* < 0.05). However, when considering that there were a majority of non-statistically significant differences when comparing the granite and limestone periods, these results should be interpreted with caution. Among children, more pronounced associations were observed during summer, although no significant differences in risk were detected between the limestone and granite periods. **Conclusions:** Although the intervention effectively lowered particle mass concentrations, only minor changes were observed in the overall epidemiological pattern. This suggests that public health improvements may be limited by factors beyond total mass reduction, such as particle mineralogy or seasonal exposure dynamics.

## 1. Introduction

This is an intervention study to follow up local actions against high levels of road dust in Visby. Visby is located on the island of Gotland in the Baltic Sea east of the southern part of the east coast of the Swedish mainland. In a previous study in Visby, the associations between daily number of patients with acute respiratory problems and the daily mean concentrations of PM_10_ were analyzed and divided into winter/spring (road dust season) and summer/autumn [1]. The associations were considerably weaker during winter/spring despite violations of 24 h limit values and high mean concentrations of PM_10_ caused by the use of limestone in asphalt and for sanding of roads with ice and snow. An overall conclusion was that the findings were in line with data, which suggests that short-term exposure to limestone is relatively harmless [1].

The exceedance of the EU and Swedish 24 h limit values for PM_10_ because of road dust gave rise to a local action program for the city, as well as changes in road maintenance by the National Road Administration. Several action strategies including modified anti-slip control, expanded road cleaning, conversion to a more durable road surface, and a reduction in the use of studded tires were developed. One strategy was to use a harder mineral for sanding icy roads by changing the use of limestone to crushed granite as an anti-slip material. Trials were carried out in 2018 using crushed granite on certain selected road sections in Visby [2], but in 2024, crushed granite began to be used for sanding on all roads, footpaths, cycle paths, and parking areas in Visby [3]. Several other action strategies have also been taken to reduce the concentrations of PM_10_. These include an expanded cleaning of the streets in Visby with the introduction of the use of vacuum cleaners in 2023 as well as a speed reduction in 2024, which may have contributed to a decrease in the concentrations of PM_10_ even during summer/autumn [4]. Chemical analysis of PM_10_ from road dust in Visby during spring 2024 revealed that calcium was the most abundant substance, the dominant element in limestone. Minor amounts of silicon, aluminum, iron, potassium, sodium, and magnesium were detected, which are dominant constituents of igneous rocks such as granite [5]. This indicates that limestone dust remains a significant contributor to PM_10_ in Visby.

PM_10_ is defined as particles with an aerodynamic diameter smaller than, or equal to, 10 µm. The short-term mortality effects associated with exposure to PM_10_ have been analyzed in several studies where a statistically significant association for all-cause mortality was found based on a meta-analysis [6]. With respect to respiratory effects, short-term exposure to PM_10_ has been shown to be associated with increases in asthma emergency room visits and hospitalizations based on a meta-analysis [7]. An increase in asthma medication sales associated with the concentrations of PM_10_ has also been shown for multiple locations in Stockholm, Sweden [8].

PM_10_ constitutes a mixture of several different components depending on origin and formation processes in the atmosphere. Based on a study performed in the Piedmont region in northwestern Italy, where PM_10_ samples were collected and analyzed from five air quality monitoring stations, amorphous carbon, nitrate salt, sulfate salt, iron oxides, and quartz were the main compounds [9]. When the composition of PM_10_ was analyzed at an urban site of Zhuozhou (a southern gateway of Beijing, China), elemental carbon, organic carbon, and water-soluble ions dominated the mass concentration. However, five trace elements (manganese, copper, arsenic, zinc, and lead) were particularly abundant, originating mainly from anthropogenic sources including transport, coal combustion, and biomass burning [10]. When focusing specifically on PM_10_ originating from road dust, a study conducted in Bragança, Portugal, where PM_10_ samples were collected with an in situ road dust sampler from eleven streets, element oxides represented the largest proportion. Very high enrichments were found for traffic-related elements including copper, zinc, sulfur, lead, and nickel [11].

With regard to the health effects related to exposure to PM_10_, size, shape, and chemical composition are factors that determine its physicochemical properties and toxicity. The health effects associated with road dust particles are not as well researched as the effects that arise from exhaust emissions from traffic [12]. However, based on a systematic literature review on the health effects associated with exposure to road dust particles, the respiratory system was found to be the most affected organ system within the human body [13].

Regarding road dust and its chemical composition, the road material and the materials used in anti-slip control are of great importance. As shown in the previous study, in analyzing the short-term respiratory effects of exposure to PM_10_ in Visby, the relative risks during winter/spring (with high content of limestone) were considerably smaller and in most cases non-significant, when compared to the relative risks during summer/autumn [1]. The lack of clear effects on the respiratory system related to high concentrations of PM_10_ during the road dust season (winter/spring) may be due to the fact that calcium carbonate can be effectively taken up by macrophages without serious effects on white blood cells or the lung mucosa [14].

However, despite the indications that limestone is relatively harmless from a health point of view, from the winter/spring of 2023/2024 onwards, winter road maintenance in Visby has switched from using limestone to using crushed granite as a measure to reduce the concentrations of PM_10_. Limestone is a sedimentary type of rock consisting of calcium carbonate (CaCO_3_). Although the previous study found no adverse respiratory effects associated with PM_10_ with high content of limestone, an in vitro study showed that A549 cells exposed to dust from calcium carbonate exhibited a slight decrease in viability as well as inflammatory reactions, although the reactions were less pronounced compared to when the corresponding cells were exposed to silicon dioxide (SiO_2_) [14].

Granite is an igneous type of rock consisting of several siliceous minerals and alkaline metal oxides. The health effects associated with exposure to granite particles have been studied among granite workers, animals, and in cell cultures. When considering workers in the granite industry, exposure to particles containing crystalline silica was associated with increased mortality from silicosis and other non-malignant respiratory diseases [15]. When hamsters were exposed to granite dust (12% quartz) and followed during a time course of 1–14 days post-exposure, there were elevated enzyme levels, excessive fluid accumulation in the lungs, increased cell numbers in the bronchoalveolar lavage fluid, as well as inhibited macrophage phagocytosis [16]. Detrimental effects were also shown when fibroblast cells from lung tissue were exposed to granite particles in different size fractions, with increases in biomarkers indicating genotoxicity, inflammation, oxidative stress, and lipid peroxidation, showing the most noticeable effects for the smallest (nano-sized) granite particles [17].

The purpose of this study was to analyze the respiratory health effects associated with short-term exposure to PM_10_ in Visby resulting from the change of anti-slip material from limestone to crushed granite and the introduction of vacuum cleaners and speed reduction. This follow-up study covers the period from 2015 to 2025 and uses the same methodology as the previously conducted study published in 2022 that analyzed the corresponding outcomes during the period from 2013 to 2019 [1].

## 2. Materials and Methods

### 2.1. Health Data

Data regarding respiratory effects including the daily number of patients with acute respiratory problems (ICD-10 diagnoses: J00–J99) seeking care at the hospital (emergency department or specialist clinic) or a primary healthcare center (including one on-call unit) were used. Data were collected from Gotland Healthcare Administration in Visby for all different care facilities in the area. The collected data also contained date of visit, patient’s age, healthcare unit, and diagnosis. The diagnostic codes were grouped into upper and lower respiratory tract diseases (J0, J3 and J1, J4, respectively) and asthma (ICD-10: J45, J46), following the same approach as our previous study in Visby [1]. The diagnosis data were also separated into the two age groups of 0–17 years and >17 years. In Table 1, the numbers of respiratory diagnoses, grouped into the above-mentioned diagnostic codes, are presented for different health care units in Visby from 2015 to 2019 (“limestone period”) and from 2023 to 2025 (“granite period”).

### 2.2. Environmental Data

**Figure 1:**
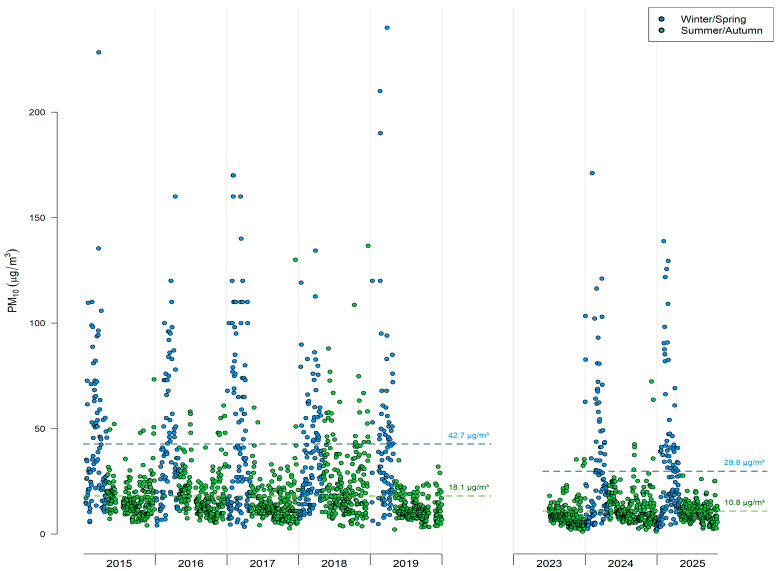
Daily mean PM_10_ concentrations (µg m^−3^) in Visby during the limestone period (2015–2019) and granite period (2023–2025). Blue dots represent winter/spring (January–April) and green dots represent summer/autumn (May–December). Dotted lines show mean values for each period, divided into winter/spring and summer/autumn.

Summary statistics on PM_10_ concentrations and meteorological variables are presented in Table 2. This includes data regarding the daily concentrations of PM_10_ during the whole year, during winter/spring, and during summer/autumn collected from Region Gotland and covered the years from 2015 to 2019 and from 1 July 2023 to 5 November 2025. Data on temperature and relative humidity for the same periods for the whole year were collected from the Swedish Meteorological and Hydrological Institute (SMHI). The PM_10_ concentrations were measured in a central part of Visby with a measurement method called IVL PModell S10 (IVL, Swedish Environmental Research Institute Ltd., Stockholm, Sweden) from 2015–2019, and with a measurement method called Fidas 200 (Palas GmbH, Karlsruhe, Germany) from 2023–2025, and daily averages of temperature and relative humidity were collected from SMHI’s weather station located at Visby Airport. Daily mean concentrations of PM_10_ are graphically presented in Figure 1. The environmental data that have been used are available at the Swedish Meteorological and Hydrological Institute (SMHI) [18].

### 2.3. Statistical Analysis

Short-term associations between daily counts of registered healthcare visits for respiratory diseases and daily mean concentrations of PM_10_ were analyzed using a Generalized Additive Model (GAM) with a quasi-Poisson distribution to account for over-dispersion. The model is specified as follows:

$$\begin{array}{cc} \log\left(E\left[Y_{t}\right]\right)=\;\beta_{0} & +\sum_{i=1}^{4}\beta_{i}({PM}_{10,t}\cdot {PV}_{i,t})+\alpha {period}_{t}+s\left({RH}_{t},\;df=4\right)+s\left({Temp}_{t},\;df=4\right)\\ & +\;s\left({day}_{t},\;df=30\right)+\gamma {DOW}_{t}+\delta {Red}_{t} \end{array}$$

where

- *E[Y_t_]* represents the expected daily number of registered respiratory visits at day *t*.
- *PM_10__,t_⋯ PV_i,t_* is an interaction term between the PM_10_ concentration and an indicator variable for the four combinations of period (limestone/granite) and season (winter/spring and summer/autumn). This allows for the estimation of four unique slope coefficients for PM_10_.
- *period_t_* is a binary variable for the two main study periods (limestone/granite) to control for the baseline shift in visit frequency.
- *s*(*RH_t_*, 4) and *s*(*Temp_t_*, 4) are penalized splines with 4 degrees of freedom (*df*) controlling for non-linear effects of relative humidity and ambient temperature, respectively.
- *s*(*day*, 30) is a penalized spline of time (observation day) to account for long-term trends and seasonality, using ~4 degrees of freedom per year.
- *DOW_t_* and *Red_t_* are factor variables for day of the week and public holidays, respectively.

The concentrations of PM_10_ were analyzed using a two-day moving average (lag01), which was also applied to temperature and relative humidity. Linear interpolation was applied to missing PM_10_ values with a maximum gap of 1 consecutive day. The effects of PM_10_ on the daily number of respiratory diagnoses were estimated assuming a linear association.

To separate the seasonal effects, an interaction variable was used to distinguish between the “winter/spring” period (January–April) and the “summer/autumn” period (May–December). The analyses were further divided into the limestone period (2015–2019) and the granite period (1 July 2023 to 5 November 2025). The years 2020–2022 were omitted from the analysis due to the atypical circumstances and healthcare-seeking patterns that prevailed during the COVID-19 pandemic. 

All statistical analyses were performed using R (version 4.1.2, The R Foundation for Statistical Computing, Vienna, Austria) with the mgcv package for Generalized Additive Models.

## 3. Results

### Relative Risks Associated with Exposure to PM_10_

The RRs associated with short-term exposure (lag01) to PM_10_ and all respiratory diagnoses, upper airways, lower airways, and asthma divided into winter/spring (January–April) and summer/autumn (May–December) and the age groups of ≤17 years and >17 years are presented in Table 3 and Table 4. These analyzes are also divided into the period where limestone was primarily used as an anti-slip material (2015–2019) and the period where crushed granite instead was used as an anti-slip material (1 July 2023 to 5 November 2025). Wald tests with *p*-values are presented to determine the differences in RRs between the periods with limestone and crushed granite.

Table 3 and Table 4 present the RRs associated with a 10 µg m^−3^ increase in PM_10_ for all respiratory diagnoses, upper airways, lower airways, and asthma divided into winter/spring and summer/autumn periods. The relative risks are divided into the period where limestone was primarily used as an anti-slip material (2015–2019) and the period where crushed granite had begun to be used as an anti-slip material (1 July 2023 to 5 November 2025). Wald tests to determine the differences in relative risks (*p* < 0.05 indicate a statistically significant difference) between the periods with limestone and crushed granite are also presented. Table 3 presents the results for children (0–17 years of age), and Table 4 presents the results for adults (>17 years of age).

**Figure 2:**
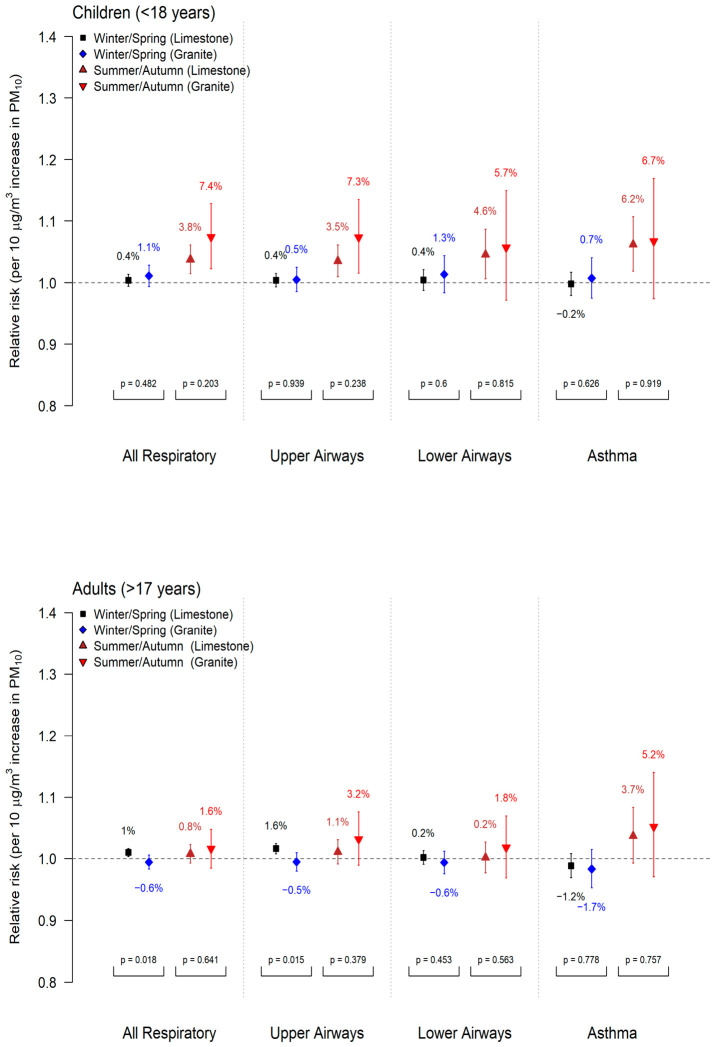
Relative risk (RR with 95% CI) of respiratory visits per 10 µg m^−3^ increase in PM_10_ (lag 0–1) during the limestone (2015–2019) and the granite (2023–2025) periods. Results are stratified by diagnostic category (all respiratory, upper airways, lower airways, and asthma), season (winter/spring and summer/autumn), and age group (children 0–17 years and adults >17 years). *p*-values indicate differences in RR between periods (Wald test).

The results presented in Figure 2, Table 3 and Table 4 clearly show that the RRs were larger during the summer/autumn period in comparison with the winter/spring period. When considering differences in effects during the limestone and granite period, the RRs were in all cases larger during the granite period with regard to children, while for adults, the RRs during the granite period were larger only during the summer/autumn period. However, statistically significant differences between the granite and the limestone period were only presented in two out of the total 16 cases, which apply to all respiratory diagnoses and upper airways for adults during winter/spring, with larger effects during the limestone period.

## 4. Discussion

### 4.1. Differences in Concentrations and Relative Risks Related to Season and the Use of Crushed Granite Versus Limestone

This follow-up study was initiated to evaluate the health effects resulting from the introduction of crushed granite as an anti-slip material, but also the effects resulting from vacuum cleaning and speed reduction. When considering the concentrations of PM_10_ in Visby during the period from 2015 to 2019 (the limestone period), the mean values were 18.1 µg m^−3^ during summer/autumn and 42.7 µg m^−3^ during winter/spring. The corresponding mean values of PM_10_ during the period from 1 July 2023 to 5 November 2025 (the granite period) were 10.8 µg m^−3^ during summer/autumn and 29.8 µg m^−3^ during winter/spring. When considering that the reductions in PM_10_ are of the same order of magnitude for both summer/autumn and winter/spring, other factors besides the introduction of crushed granite must have played a major role. These include an expanded cleaning of the streets in Visby with the introduction of the use of vacuum cleaners in 2023 as well as a speed reduction in 2024, which may have contributed to a decrease in the concentrations of PM_10_ even during summer/autumn [4].

When considering the differences in relative risks with regard to the investigated time periods, the time period from 2015 to 2019 constitutes an indicator of the conditions that prevailed before crushed granite was introduced as an anti-slip material, and the time period from 2023 to 2025 constitutes an indicator of the conditions that prevailed after crushed granite was introduced. The time period from 2020 to 2022 was omitted due to the special circumstances that prevailed during the COVID-19 pandemic.

When comparing the point estimates of the relative risks (Wald test) with respect to the time periods of 2015–2019 (limestone) and 2023–2025 (granite), only 2 out of 16 were considered statistically significantly different. The RRs for adults’ all respiratory visits and adults’ upper airway visits were significantly smaller during the granite period. For children, there was a tendency that RRs were larger during the granite period. For adults, the RRs during winter/spring tended to be smaller during the granite period in comparison with the limestone period, while the reverse relationship prevailed during summer/autumn.

Based on all the RRs presented in Figure 2, Table 3 and Table 4, statistically significant associations are only shown for eight RRs, which corresponds to a quarter of the total number of 32 RRs. For children, all six statistically significant associations are shown during summer/autumn, which corresponds to the time period where particle concentrations originating from anti-slip materials were much lower in comparison with winter/spring. For adults, there are only two statistically significant associations, which are both presented for the limestone period during winter/spring.

Due to a relatively smaller amount of data with respect to the time period from 2023 to 2025 in comparison with the time period from 2015 to 2019, the confidence intervals of the RRs were wider during the granite period.

### 4.2. Possible Reasons for the Results

Based on the Wald tests, in analyzing the differences in RRs between the limestone and the granite period, statistically significant differences are presented in two out of the total 16 cases, which are “all respiratory diagnoses” and “upper airways” for adults during winter/spring, with *p*-values of 0.018 and 0.015, respectively. However, given the multiple comparisons (16 tests at α = 0.05), these findings are marginally above chance expectation and should be interpreted with caution. Apart from these two cases, Wald tests indicated no significant differences in PM_10_ effects between these two periods. Moreover, the RRs were in general larger during summer/autumn, which corresponds to the period when PM_10_ consists of road dust to a relatively very small extent. This is similar to the previous study [1], based on the period from 2013 to 2019. The introduction of crushed granite during 2023–2025 has not significantly changed this situation, despite its potentially more toxic nature.

However, when the chemical composition of PM_10_ from road dust was analyzed in Visby during springtime 2024, it turned out that calcium was the most abundant substance, which is the dominant element in limestone. Relatively smaller amounts of silicon, aluminum, iron, potassium, sodium, and magnesium were found, which constitute dominant substances in igneous rocks such as granite [5]. It is possible that crushed granite causes increased wear on tires and road surfaces, especially on road and parking areas that have a significant proportion of limestone material in the asphalt [19]. Consequently, although crushed granite began to be used in 2023, limestone still accounts for a dominant share of PM_10_ that the population of Visby is exposed to during winter/spring. Therefore, the lack of statistically significant differences in the RRs between the limestone and the granite period may be due to the fact that the change in the chemical composition of exposure to PM_10_ is actually small and that the potentially more toxic content in crushed granite does not reach such high concentrations where it can have any impact on the relative risks.

When considering the differences in the RRs between winter/spring and summer/autumn, they were generally larger and more consistent during summer/autumn. The centrally located measuring station in Visby becomes less representative for the exposure of locally generated PM_10_ originating from road dust for the population outside of its immediate area, which is most prominent during winter/spring. The healthcare units in Visby also receive visitors from areas outside Visby. During summer/autumn, when locally generated road dust is not as prominent, the measuring station also becomes more representative for the areas outside Visby’s city center when the long-range transported particles dominate.

Another possible reason for the relatively smaller and less consistent RRs during winter/spring is that the concentrations at the measuring station are less representative of exposure during winter/spring due to relatively less time spent outdoors. Based on a review and meta-analysis analyzing outdoor PM infiltration into indoor environments in Northern, Central and Southern Europe, the infiltration rate of PM_10_ was in the range of 0.26–0.47 and showed higher values during summertime in comparison with wintertime [20]. If the same conditions are assumed to prevail in Visby, the measured concentrations of PM_10_ at the centrally located measuring station will be less representative during winter/spring in comparison with summer/autumn.

Visby’s significant seasonal population fluctuations due to tourism may also influence the findings. A proportion of respiratory visits during summer likely originates from non-residents, potentially affecting risk estimates. Unlike the winter months, when the population consists almost entirely of permanent residents, the denominator of the population at risk increases dynamically during the peak tourist season.

Furthermore, comparing the baseline (2015–2019) and follow-up (2023–2025) periods requires consideration of the COVID-19 pandemic’s impact. Beyond altering actual disease patterns, the pandemic may have shifted healthcare-seeking behavior regarding respiratory symptoms.

### 4.3. Policy Implications and Future Research Needs

From a policy point of view, the introduction of crushed granite has decreased the concentrations of PM_10_, but the RRs have not changed significantly as a result of this introduction. Consequently, the use of crushed granite as an anti-slip material has not been shown to worsen the short-term respiratory health effects in Visby. However, the uncertainties in the exposure assessment would need to be addressed in future research. Instead of using only one centrally located measuring station, multiple measuring stations would be needed to assess the spatial resolution of locally generated road dust particles and where relative risks for short-term respiratory effects are calculated for more geographically specific areas. Moreover, the long-term health effects associated with exposure to PM_10_ containing crushed granite would also need to be investigated, which are not presented in this study design with time-series analysis.

### 4.4. Strengths and Limitations of This Study

A strength of this study is the use of extensive register data on healthcare visits from all relevant healthcare units in Visby, combined with continuous measurements of air pollution, which enables comparisons between health effects during the limestone and the granite period.

A limitation of this study is that PM_10_ is the only air pollutant that was measured over the study period, and no multi-pollutant models with other air pollutants or pollen have been possible to conduct. This means that the results could be confounded by other air pollutants and by pollen during spring and summer, which can also affect the respiratory system. Additionally, misclassification of exposure due to the use of a single centrally located monitoring station is a common limitation in ecological studies of short-term air pollution effects. During winter/spring in Visby, this measurement limitation could be particularly relevant, as locally generated PM_10_ from road dust (including sanding) likely represents a considerable share of the measured concentrations. Consequently, exposure misclassification may be more pronounced during winter/spring, making seasonal comparisons of relative risks less certain. Furthermore, detailed information about the chemical composition of the particles is lacking over the entire study period, which makes it difficult to more accurately attribute the source of the particle concentrations.

## 5. Conclusions

An overall conclusion of this study is that the short-term respiratory health effects associated with exposure to PM_10_ have not changed significantly since the introduction of crushed granite as an anti-slip material in 2023. The decline in the PM_10_ concentrations when comparing the limestone period (2015–2019) and the granite period (1 July 2023 to 5 November 2025) is of the same order of magnitude for both winter/spring and summer/autumn. This indicates that the other measures in terms of vacuum cleaning of the streets and speed reduction have been effective in reducing the concentrations of PM_10_ in Visby in addition to the introduction of crushed granite.

The relative risks were generally larger during summer/autumn compared to winter/spring. This may reflect differences in exposure measurement accuracy between seasons. During summer/autumn, when PM_10_ consists primarily of regional particles, the centrally located measuring station provides representative exposure assessment for the population. In contrast, during winter/spring, when locally generated road dust dominates PM_10_ concentrations, the single measurement location cannot adequately capture spatial exposure variation, leading to exposure misclassification and reduced ability to detect respiratory health effects.

## Figures and Tables

**Table 1:** The numbers of respiratory diagnoses at different healthcare units in Visby during the period from 2015 to 2019 (limestone) and 1 July 2023 to 5 November 2025 (granite).

Number and *n*/Day	Adults (18+) Limestone (15–19)	Adults (18+) Granite (23–25)	Children (0–17) Limestone (15–19)	Children (0–17) Granite (23–25)
Number of days	1826	859	1826	859
All respiratory (ICD-10: J00–J99)	39,272	18,553	17,883	7436
All respiratory (average per day)	21.5	21.6	9.8	8.7
Upper airways (ICD-10: J0, J3)	21,061	9450	11,663	4814
Upper airways (average per day)	11.5	11.0	6.4	5.6
Lower airways (ICD-10: J1, J4)	13,698	6859	5701	2357
Lower airways (average per day)	7.5	8.0	3.1	2.7
Asthma (ICD-10: J45, J46)	4200	2336	4866	2014
Asthma (average per day)	2.3	2.7	2.7	2.3

**Table 2:** Summary statistics of PM_10_ (µg m^−3^) in Visby during the limestone period from 2015 to 2019, and the granite period from 1 July 2023 to 5 November 2025, divided into summer/autumn and winter/spring.

Period	Season	Number of Days with Observations	Number of Days with Missing Observations	Mean (µg m^−3^)	Median (µg m^−3^)	Minimum–Maximum (µg m^−3^)
Limestone (15–19)	Summer/Autumn	1 073	152	18.1	14.4	2.2–136.6
Limestone (15–19)	Winter/Spring	496	105	42.7	31.8	3.5–248.63
Granite (23–25)	Summer/Autumn	618	0	10.8	9.2	1.2–72.4
Granite (23–25)	Winter/Spring	232	9	29.8	21.4	2.3–171.1

**Table 3:** The relative risks associated with a 10 µg m^−3^ increase in PM_10_ for children at lag01 during the limestone period (2015–2019) and the granite period (1 July 2023 to 5 November 2025) divided into summer/autumn and winter/spring.

Diagnoses (Children, 0–17 Years)	Season	Period	RR	Lower 95% CI	Upper 95% CI	*p*-Value	*p*-Value Wald Difference
All respiratory diagnoses	Winter/Spring	Limestone	1.004	0.994	1.014	0.426	–
All respiratory diagnoses	Winter/Spring	Granite	1.011	0.994	1.029	0.213	0.482
All respiratory diagnoses	Summer/Autumn	Limestone	1.038	1.015	1.061	0.001	–
All respiratory diagnoses	Summer/Autumn	Granite	1.074	1.023	1.128	0.004	0.203
Upper airways	Winter/Spring	Limestone	1.004	0.993	1.015	0.460	–
Upper airways	Winter/Spring	Granite	1.005	0.986	1.025	0.617	0.939
Upper airways	Summer/Autumn	Limestone	1.035	1.010	1.061	0.007	–
Upper airways	Summer/Autumn	Granite	1.073	1.015	1.135	0.013	0.238
Lower airways	Winter/Spring	Limestone	1.004	0.988	1.021	0.615	–
Lower airways	Winter/Spring	Granite	1.013	0.984	1.044	0.380	0.600
Lower airways	Summer/Autumn	Limestone	1.046	1.006	1.087	0.023	–
Lower airways	Summer/Autumn	Granite	1.057	0.972	1.150	0.196	0.815
Asthma	Winter/Spring	Limestone	0.998	0.980	1.017	0.832	–
Asthma	Winter/Spring	Granite	1.007	0.975	1.041	0.663	0.626
Asthma	Summer/Autumn	Limestone	1.062	1.019	1.107	0.005	–
Asthma	Summer/Autumn	Granite	1.067	0.974	1.169	0.163	0.919

**Table 4:** The relative risks associated with a 10 µg m^−3^ increase in PM_10_ for adults at lag01 during the limestone period (2015–2019) and the granite period (1 July 2023 to 5 November 2025) divided into summer/autumn and winter/spring.

Diagnoses (Adults, >17 Years)	Season	Period	RR	Lower 95% CI	Upper 95% CI	*p*-Value	*p*-Value Wald Difference
All respiratory diagnoses	Winter/Spring	Limestone	1.010	1.003	1.016	0.003	–
All respiratory diagnoses	Winter/Spring	Granite	0.994	0.983	1.005	0.311	0.018
All respiratory diagnoses	Summer/Autumn	Limestone	1.008	0.993	1.023	0.323	–
All respiratory diagnoses	Summer/Autumn	Granite	1.016	0.985	1.048	0.322	0.641
Upper airways	Winter/Spring	Limestone	1.016	1.007	1.025	<0.001	–
Upper airways	Winter/Spring	Granite	0.995	0.980	1.010	0.474	0.015
Upper airways	Summer/Autumn	Limestone	1.011	0.991	1.031	0.286	–
Upper airways	Summer/Autumn	Granite	1.032	0.989	1.076	0.144	0.379
Lower airways	Winter/Spring	Limestone	1.002	0.991	1.013	0.753	–
Lower airways	Winter/Spring	Granite	0.994	0.976	1.012	0.496	0.453
Lower airways	Summer/Autumn	Limestone	1.002	0.977	1.027	0.896	–
Lower airways	Summer/Autumn	Granite	1.018	0.969	1.070	0.483	0.563
Asthma	Winter/Spring	Limestone	0.988	0.969	1.008	0.250	–
Asthma	Winter/Spring	Granite	0.983	0.953	1.015	0.295	0.778
Asthma	Summer/Autumn	Limestone	1.037	0.993	1.083	0.103	–
Asthma	Summer/Autumn	Granite	1.052	0.970	1.140	0.220	0.757

## Data Availability

Health registry data can be requested from the Gotland Health Care Administration (https://gotland.se/vard-och-halsa, last access 6 June 2026). Air pollution data and meteorological data can be requested from the Swedish Meteorological and Hydrological Institute (SMHI) (https://www.smhi.se/data, last access 6 June 2026).

## Abbreviations

The following abbreviations are used in this manuscript:

A549	A type of human alveolar basal epithelial cell
CaCO_3_	Calcium carbonate
ERD	Emergency department
ICD	International Classification of Diseases
PM_10_	Particles with an aerodynamic diameter smaller than, or equal to, 10 µm
RR	Relative risk
SiO_2_	Silicon dioxide
